# Response to: Comment on “Choroidal Thickness in Patients with Mild Cognitive Impairment and Alzheimer's Type Dementia”

**DOI:** 10.1155/2016/2898704

**Published:** 2016-11-29

**Authors:** Mehmet Bulut, Aylin Yaman, Muhammet Kazim Erol, Fatma Kurtuluş, Devrim Toslak, Berna Doğan, Deniz Turgut Çoban, Ebru Kaya

**Affiliations:** ^1^Ophthalmology Department, Antalya Training and Research Hospital, Antalya, Turkey; ^2^Neurology Department, Antalya Training and Research Hospital, Antalya, Turkey; ^3^Biometry and Genetics Unit, Department of Animal Science, Faculty of Agriculture, Akdeniz University, Antalya, Turkey

We would like to thank Ilhan et al. [1] for their comments regarding our article. In our study, we found out that choroidal thickness (CT) decreased in the eyes of patients with both Alzheimer's type dementia (ATD) and mild cognitive impairment (MCI) compared to the healthy control group. Therefore, we suggested that CT value could be used as a new biomarker in early diagnosis of ATD and MCI patients and follow-up of their progression [2].

We performed statistical analysis using the values of single eyes and both eyes in our study. We found statistically significant results for both single eyes and both eyes, which was consistent with the suggestion of Ilhan et al. [1]. For that reason, we preferred using the values of both eyes in order to extend the number of data in our paper. We also found out in our study that both parapapillary retinal nerve fiber layer (RNFL) and macular ganglion cell-inner plexiform layer (GC-IPL) thickness decreased in MCI and ATD patients compared to the healthy control group. However, we did not use these data in our paper because we wanted to use them in another study for an extended group of participants. We think that the pathology of central nervous system in ATD and MCI patients may affect both RNFL and GC-IPL in eyes at an early stage. There are publications in the literature relating to this matter [3, 4].

In our study, we found a positively significant correlation between the mini mental state examination (MMSE) test score and CT values, whereas these findings were obtained before the participants were assigned to the groups and we used these data in our paper. We also analyzed the correlation in each group. However, we could not find any statistically significant correlation. This might be associated with the low number of data in each group.
